# Evaluation of Glutathione in Spike Protein of SARS-CoV-2 Induced Immunothrombosis and Cytokine Dysregulation

**DOI:** 10.3390/antiox13030271

**Published:** 2024-02-22

**Authors:** Brandon Norris, Abraham Chorbajian, John Dawi, Aishvaryaa Shree Mohan, Ira Glassman, Jacob Ochsner, Yura Misakyan, Arbi Abnousian, Anthony Kiriaki, Kayvan Sasaninia, Edith Avitia, Cesar Ochoa, Vishwanath Venketaraman

**Affiliations:** College of Osteopathic Medicine of the Pacific, Western University of Health Sciences, Pomona, CA 91766, USA; brandon.norris@westernu.edu (B.N.); abraham.chorbajian@westernu.edu (A.C.); john.dawi@westernu.edu (J.D.); amohan@westernu.edu (A.S.M.); ira.glassman@westernu.edu (I.G.); jacob.ochsner@westernu.edu (J.O.); yura.misakyan@westernu.edu (Y.M.); arbi.abnousian@westernu.edu (A.A.); anthony.kiriaki@westernu.edu (A.K.); kayvan.sasaninia@westernu.edu (K.S.); eavitia@westernu.edu (E.A.); cochoa@westernu.edu (C.O.)

**Keywords:** glutathione, COVID-19, oxidative stress, immunothrombosis

## Abstract

Thrombotic microangiopathy has been identified as a dominant mechanism for increased mortality and morbidity in coronavirus disease 2019 (COVID-19). In the context of severe COVID-19, patients may develop immunothrombosis within the microvasculature of the lungs, which contributes to the development of acute respiratory distress syndrome (ARDS), a leading cause of death in the disease. Immunothrombosis is thought to be mediated in part by increased levels of cytokines, fibrin clot formation, and oxidative stress. Glutathione (GSH), a well-known antioxidant molecule, may have therapeutic effects in countering this pathway of immunothrombosis as decreased levels of (GSH) have been associated with increased viral replication, cytokine levels, and thrombosis, suggesting that glutathione supplementation may be therapeutic for COVID-19. GSH supplementation has never been explored as a means of treating COVID-19. This study investigated the effectiveness of liposomal glutathione (GSH) as an adjunctive therapy for peripheral blood mononuclear cells (PBMC) treated with SARS CoV-2 spike protein. Upon the addition of GSH to cell cultures, cytokine levels, fibrin clot formation, oxidative stress, and intracellular GSH levels were measured. The addition of liposomal-GSH to PBMCs caused a statistically significant decrease in cytokine levels, fibrin clot formation, and oxidative stress. The addition of L-GSH to spike protein and untreated PBMCs increased total intracellular GSH, decreased IL-6, TGF-beta, and TNF-alpha levels, decreased oxidative stress, as demonstrated through MDA, and decreased fibrin clot formation, as detected by fluorescence microscopy. These findings demonstrate that L-GSH supplementation within a spike protein-treated PBMC cell culture model reduces these factors, suggesting that GSH supplementation should be explored as a means of reducing mediators of immunothrombosis in COVID-19.

## 1. Introduction

Immunothrombosis has been identified as a key pathologic component of COVID-19 found in patients with severe and long COVID-19 who display amyloid fibrin microclots within microvessels [1,2]. Immunothrombosis in COVID-19 is induced by the interconnection between the hyperactivation of the host’s innate immune system and the host’s natural coagulation processes. Research has shown that severe COVID-19 induces a macrophage activation syndrome (MAS)-like event, resulting in cytokine storm, immune cell recruitment, and subsequent endothelial damage [3]. This process yields an excess of reactive oxygen species (ROS), which impairs endothelial function, reduces the bioavailability of nitric oxide (NO), and triggers the release of tissue factor (TF), cumulatively contributing to the formation of microclots in the pulmonary vasculature [3,4]. It is believed that these microclots contribute to both thromboembolic events seen in severe COVID-19 patients and the prolonged symptoms seen in patients with long COVID-19 [5].

There is currently no known effective treatment for long COVID [6,7]. A meta-analysis found that 43% of patients had long COVID at >/= 28 days from infection [8]. Current treatments for COVID-19 include antivirals nirmaltrelvir/ritonavir, remdesivir, and baricitinib. A long-term follow-up on the SOLIDARITY Finland trial assessing the efficacy of remdesivir in COVID-19 found no statistically significant evidence of remdesivir’s benefits in long COVID symptoms [9]. A recent observational study found that, in a group of vaccinated, non-hospitalized patients, oral nirmatrelvir/ritonavir was not associated with reduced long COVID symptoms beyond 90 days post-infection [10]. Baricitinib is currently undergoing a clinical trial to assess efficacy against long COVID, referred to as REVERSE-LC, with results expected in 2030. Given the climate of therapeutics for preventing and treating long COVID, more research is needed to explore primary COVID-19 treatments and adjunctive treatments to improve morbidity and mortality. Research into reducing inflammation and platelet aggregation has been explored via the utilization of aspirin, which has shown a beneficial effect as an adjunctive treatment in hospitalized COVID-19 patients [11].

Glutathione (GSH), an antioxidant found ubiquitously, has been linked to pulmonary inflammation, COVID-19, and thrombosis. GSH is utilized by glutathione peroxidase (GPX) to reduce ROS. Activated platelets can produce ROS through NADPH oxidase signaling, resulting in the forward-feeding cascade of repeated ROS production and platelet activation and recruitment seen in COVID-19. Recent studies have identified a negative correlation between GSH and levels of Interleukin-6 (IL-6) and transforming growth factor-β (TGF-β). The SARS-CoV-2 viral N protein has been found to increase the synthesis of IL-6, which has been linked to the downregulation of glutamate cysteine ligase (GCL), the rate-limiting enzyme in GSH synthesis [12,13]. IL-6 is a proinflammatory cytokine that also causes oxidative stress and systemic inflammation [14]. TGF-β, another cytokine elevated in COVID-19, also functions to downregulate GCL expression [15,16]. TGF-β is an anti-inflammatory cytokine, elevated in inflammatory conditions such as HIV, that has demonstrated potent reduction in GSH synthesis when overexpressed, resulting in increased ROS [17,18]. GSH has also been shown to reduce levels of TNF-α, a proinflammatory cytokine found upregulated in COVID-19 which triggers cytokine release syndrome (CRS) and facilitates SARS-CoV-2 binding with angiotensin-converting enzyme 2 (ACE2) [19,20]. Lastly, GSH has a negative association with the lipid peroxide byproduct malondialdehyde, which is elevated in patients with COVID-19 [21,22]. 

Research has found that GSH can inhibit human platelet aggregation, suggesting that exogenous supplementation with GSH may inhibit platelet activation [23,24]. Research has also shown that COVID-19 decreases GSH synthesis by decreasing GSH precursor cystine uptake, increasing the efflux of thiols, inhibiting nuclear factor-erythroid 2 p45-related factor (Nrf2), and inhibiting BRCA1 [25,26,27]. Decreased GSH leads to a cascade of events, including an inability to mitigate oxidative stress, a subsequent increase in the release of cytokines IL-6 and TGF-β, both of which are shown to further decrease GSH synthesis, and an increase in platelet activation, contributing to microclot formation. As a mediator of ROS and platelet activation, therapies which serve to increase levels of GSH may serve a protective role in both acute and long COVID-19. 

In this study, we hypothesize that liposomal GSH supplementation will result in increased total GSH, a reduction in cytokines associated with GSH synthesis reduction, and ultimately a reduction in fibrin clot formation induced by COVID-19 spike protein. Human peripheral blood mononuclear cells (PBMCs) are a type of blood cell with a round nucleus, which is isolated from peripheral blood. These cells contain lymphocytes, monocytes, dendritic cells, and natural killer cells. In this study, peripheral blood mononuclear cells (PBMCs) from healthy human volunteers were either untreated, treated with COVID-19 spike protein (SP), treated with liposomal GSH (L-GSH), or treated with both COVID-19 spike protein and L-GSH. The cultures were then terminated at 1 h, 3 days, and 7 days post-treatment, after which total GSH levels, malondialdehyde levels, and cytokine profiles were assessed. In addition, cell culture supernatants were assessed for microclot formation post-treatment. We utilized thioflavin T stain and fluorescence microscopy to visualize microclot formation and quantified the results using Image-J software. The findings of this study may offer new insights into treatment modalities against immunothrombosis induced by SARS-CoV2 infection.

## 2. Materials and Methods

### 2.1. Subject Recruitment

Healthy patients between the ages of 18 and 65 were recruited at the Patient Care Center at Western University of Health Sciences. Participants were evaluated with a physical exam and blood was drawn by a physician to assess eligibility for the study. Blood samples were subjected to a complete metabolic panel, a pregnancy test (if applicable), and an assessment for hepatitis antibodies, HbA1C levels, and HIV antibodies. The exclusion criteria involve conditions or lifestyle behaviors that result in abnormalities of liver function tests, which can be independently responsible for depleted glutathione levels or inflammation. Patients with a history of chronic hepatitis B or hepatitis C virus infection, HIV/AIDS, TB, uncontrolled T2DM, SARS-CoV2 infection within 6 months, and a history of alcohol abuse within 6 months were excluded. Study participants meeting the inclusion criteria (*n* = 11) had blood drawn for the extraction of peripheral blood mononuclear cells (PBMCs) and plasma to be used in the study. 

### 2.2. Isolation and Culture of Peripheral Blood Mononuclear Cells

The isolation of PBMC and plasma from the collected blood samples was achieved by density centrifugation using ficoII histopaque (Sigma, St. Louis, MO, USA). Blood was gently placed on top of ficoII histopaque at a 1:1 ratio and centrifuged at 1800 rpm and 25 °C. Plasma and PBMC layers were aspirated and collected in microcentrifuge tubes. PBMCs were washed and centrifuged with isotonic PBS 3 times before being resuspended in RPMI with 20% autologous plasma. The resultant cell suspension (10^6^ cells/mL) was loaded in a poly-L-lysine-coated 96-well culture plate and incubated at 37 °C with 5% CO_2_ overnight to allow for monocyte adherence. 

### 2.3. Treatment of Peripheral Blood Mononuclear Cells with Spike Protein (SP) and Liposomal Glutathione (L-GSH)

**Figure 1:**
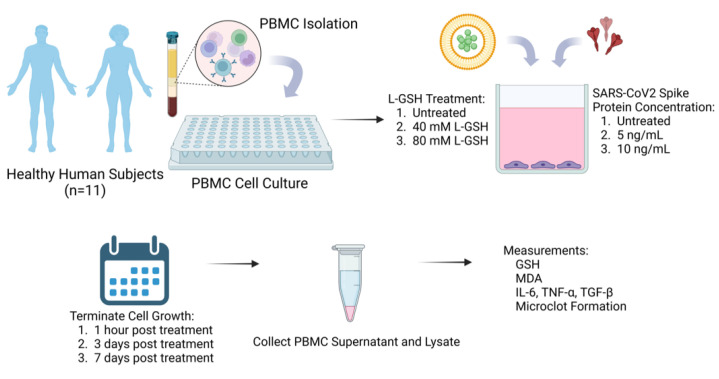
Schematic of study methods, where PBMCs were cultured from healthy subjects and treated with no L-GSH, 40 mM L-GSH, or 80 mM L-GSH, in addition to no spike protein, 5 ng/mL spike protein, or 10 ng/mL spike protein. Cell cultures were terminated at 1 h, 3 days, or 7 days post-treatment, and cell culture media and centrifuged pellets were then used for assays, including GSH, MDA, cytokine ELISA, and microclot fluorescent studies.

L-GSH was provided by our collaborator, Dr. Guilford, from Your Energy Systems Inc., Palo Alto, CA, USA. SARS-CoV2 nucleocapsid/spike protein (RBD) recombinant protein (Cat # RP-87706) was procured from ThermoFisher Scientific (Thermofisher Scientific, Waltham, MA, USA). The SP original stock concentration was 1.5 mg/mL. This was stored in −20 °C and diluted to experimental concentrations utilizing an aseptic technique in a biosafety cabinet. L-GSH and SP were applied to each well alone or in combination at the following concentrations: no L-GSH, 40 mM L-GSH, 80 mM L-GSH, no SP, 5 ng/mL SP, and 10 ng/mL SP. Spike protein concentrations were chosen based on a previous study with spike protein and thioflavin T-mediated fluorescence microscopy, where 1 ng/mL was shown to be sufficient for imaging spike protein-induced clot formation [28]. Treated PBMCs were incubated at 37 °C at 5% CO_2_ until the termination of growth 1 h, 3 days, and 7 days post-treatment. Timepoints selected as PBMC cultures were only viable within the same media for 7 days, and so this provided the maximal amount of time to measure our variables. A portion of the supernatant was utilized for microscopy. The remaining supernatant was collected in microcentrifuge tubes and stored at −80 °C for later cytokine assessment. Adherent PBMCs were lysed with cold sterile nanopure water, collected in microcentrifuge tubes, and stored at −80 °C for the subsequent assessment of relevant biomarkers. Figure 1 demonstrates a visual schematic of this process.

### 2.4. Glutathione Level Quantification

Total glutathione from PBMC lysates derived from healthy subjects was measured post-SP and post-L-GSH treatment using the GSH colorimetric kit from Arbor Assays (Catalog No. K006-H1) following the protocol provided by the manufacturer (Arbor Assays, Ann Arbor, MI, USA). Total GSH levels were normalized against total PBMC protein and reported as µM GSH/µg protein. 

### 2.5. Malondialdehyde Level Quantification

Malondialdehyde (MDA) is a lipid peroxidation byproduct and marker for oxidative stress. MDA levels from untreated, SP- and L-GSH-treated PBMC lysates were measured via the Thiobarbituric Acid Reactive Substances (TBARS) assay kit from Cayman Chemicals (Item No. 10009055) following the protocol provided by the manufacturer. MDA levels were normalized against the total protein in PBMCs and reported as µM MDA/µg protein.

### 2.6. Assessment of Cytokine Levels

Cytokine profiles from the untreated, SP-, and L-GSH-treated PBMC supernatants were assessed via the sandwich enzyme-linked immunosorbent assay (ELISA). TNF-α levels were measured via a TNF-α human uncoated ELISA kit (Cat # 88-7346-88). IL-6 levels were measured using the IL-6 human uncoated ELISA kit (Cat # 88-7066-99). TGF-β levels were measured using the TGF-β 1 human/mouse uncoated ELISA Kit (Cat # 88-8350-88). All ELISA kits were procured from Thermofisher Scientific and cytokine levels were determined following the manufacturer’s protocol (Thermofisher Scientific, Waltham, MA, USA). All cytokines were normalized against total proteins in PBMCs supernatant and reported as pg cytokine/µg protein.

### 2.7. Assessment of Microclot Formation with Thioflavin T Stain and Fluorescence Microscopy

Overall, 20 microliters of cell culture with a 5 micromolar concentration of thioflavin T were dropped onto microscope slides and then fixed in 3.8% formaldehyde and PBS. Thioflavin T binds to amyloid fibrin clots and allows them to be visualized through fluorescence microscopy (Pretorius studies reference). When visualizing microclot formations using the fluorescent microscope, the thioflavin T excitation wavelength was 450–488 nm, and the emission wavelength was from 499 to 529 mm. To quantify microclot formation, we utilized microscopic visualization and the measurement of thioflavin fluorescence intensity using Image-J software in human PBMC at 1 h, 3-day, and 7-day intervals.

### 2.8. Statistical Analysis

Statistical analysis was performed using GraphPad Prism Software version 10. A comparison between the two treatment categories was conducted via the Mann–Whitney Test. Comparison between three or more treatment categories was performed using one-way ANOVA. A *p*-value < 0.05 is considered statistically significant. 

## 3. Results

### 3.1. Total Glutathione Levels Elevated after Liposomal Glutathione Supplementation in Human Peripheral Blood Mononuclear Cells

**Figure 2:**
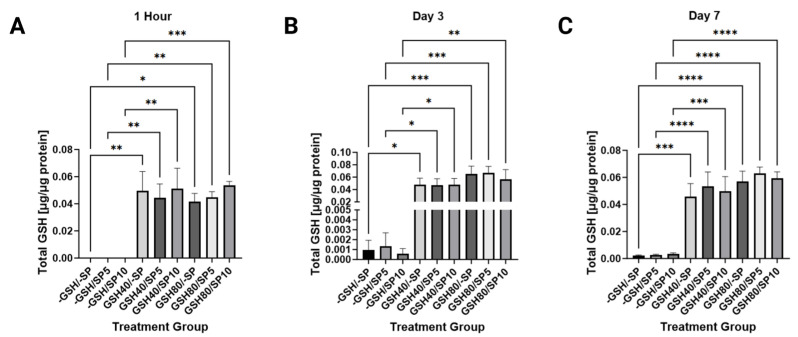
Total GSH levels in human PBMCs normalized against total PBMC protein levels. (**A**) Total GSH levels were measured in untreated human PBMCs, PBMCs treated with either 5 ng/mL spike protein (SP), 10 ng/mL SP, 40 mM liposomal GSH (L-GSH), 40 mM L-GSH plus 5 ng/mL SP, 40 mM L-GSH plus 10 ng/mL SP, 80 mM L-GSH, 80 mM L-GSH plus 5 ng/mL SP, or 80 mM L-GSH plus 10 ng/mL SP at 1 h post-treatment; (**B**) total GSH levels were measured in the same groups as Figure 2A at 3 days post-treatment; (**C**) total GSH levels were measured in the same groups as Figure 2A at 7 days post-treatment. Comparisons were made using one-way ANOVA. Statistically significant *p*-values are indicated by asterisks, and *p*-values < 0.05 (*), <0.01 (**), <0.001 (***), and <0.0001 (****) are considered significant.

Total GSH levels in PBMCs were measured with two different concentrations of GSH, 40 mM or 80 mM, within untreated groups, PBMCs treated with 5 ng/mL of spike protein, and PBMCs treated with 10 ng/mL of spike protein. There was a significant increase in total GSH in 1 h, 3 days, and 7 days when PBMCs were treated with either a 40 mM or 80 mM L-GSH, regardless of the spike protein concentration added (Figure 2).

### 3.2. Liposomal Glutathione Supplementation Reduced Interleukin-6, Transforming Growth Factor-B, and Tumor Necrosis Factor-a in Human Peripheral Blood Mononuclear Cells

**Figure 3:**
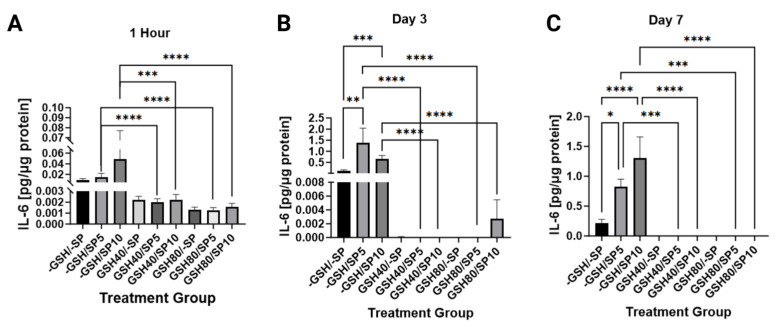
Measurements of IL-6 levels in human PBMCs normalized against total protein. (**A**) IL-6 levels were measured in untreated human PBMCs, PBMCs treated with either 5 ng/mL spike protein (SP), 10 ng/mL SP, 40 mM liposomal GSH (L-GSH), 40 mM L-GSH plus 5 ng/mL SP, 40 mM L-GSH plus 10 ng/mL SP, 80 mM L-GSH, 80 mM L-GSH plus 5 ng/mL SP, or 80 mM L-GSH plus 10 ng/mL SP at 1 h post-treatment; (**B**) total IL-6 levels were measured in the same groups as Figure 3A at 3 days post-treatment; (**C**) total IL-6 levels were measured in the same groups as Figure 3A at 7 days post-treatment. Statistically significant *p*-values are indicated by asterisks, and *p*-values < 0.05 (*), <0.01 (**), <0.001 (***), and <0.0001 (****) are considered significant.

IL-6 was measured between L-GSH and spike protein in both treated and untreated PBMCs. A significant decrease in IL-6 count was noted on day 1 and day 3 when either 40 mM L-GSH or 80 mM L-GSH treatment was combined with 5 ng/mL of spike protein (*p* < 0.0001). This significant relationship was also seen (*p* < 0.001) on day 7. A significant decrease in IL-6 count was observed when either a 40 mM (*p* < 0.001) or 80 mM (*p* < 0.0001) of L-GSH treatment was combined with 10 ng/mL of spike protein on day 1. This was sustained into day three and day seven with significance levels of (*p* < 0.0001) for both concentrations of L-GSH. There was a significant increase in IL-6 noted with the use of spike protein alone at 5 ng/mL and 10 ng/mL at day 3 (*p* < 0.01) (*p* < 0.001) and day seven (*p* < 0.05) (*p* < 0.0001), respectively (Figure 3).

**Figure 4:**
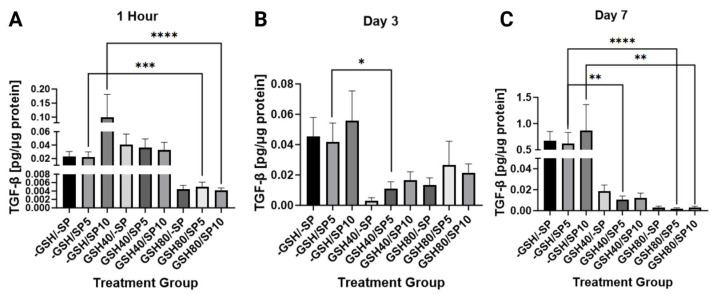
Measurements of TGF-β levels in human PBMCs normalized against total protein. (**A**) TGF-β levels were measured in untreated human PBMCs, PBMCs treated with either 5 ng/mL spike protein (SP), 10 ng/mL SP, 40 mM liposomal GSH (L-GSH), 40 mM L-GSH plus 5 ng/mL SP, 40 mM L-GSH plus 10 ng/mL SP, 80 mM L-GSH, 80 mM L-GSH plus 5 ng/mL SP, or 80 mM L-GSH plus 10 ng/mL SP at 1 h post-treatment; (**B**) total TGF-β levels were measured in the same groups as Figure 4A at 3 days post-treatment; (**C**) total TGF-β levels were measured in the same groups as Figure 4A at 7 days post-treatment. Statistically significant *p*-values are indicated by asterisks, and *p*-values < 0.05 (*), <0.01 (**), <0.001 (***), and <0.0001 (****) are considered significant.

The relationship of TGF-β with spike protein was monitored in treated and untreated PBMCs. TGF-β levels were significantly decreased on day one, when 80 mM of L-GSH was combined with 5 ng/mL of spike protein (*p* < 0.001) and 10 ng/mL of spike protein (*p* < 0.0001). On day three, a significant decrease in TGF-β levels was noted with a 40 mM supplementation of L-GSH to 5 ng/mL of spike protein (*p* < 0.05). A sustained significant decrease was noted on day 7 when either 40 mM (*p* < 0.01) or 80 mM (*p* < 0.0001) of L-GSH was combined with 5 ng/mL of spike protein. This was also observed when 80 mM of L-GSH treatment was added to 10 ng/mL of spike protein (*p* < 0.01) (Figure 4). 

**Figure 5:**
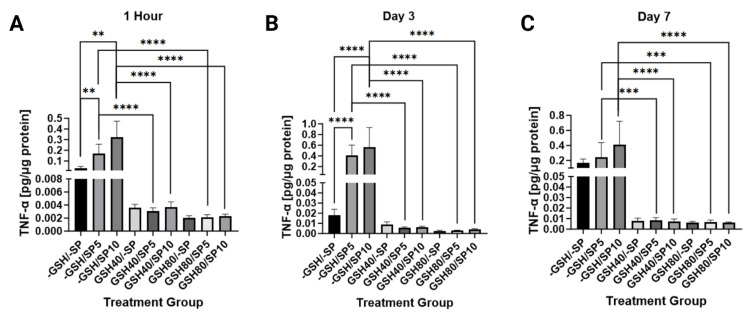
Measurements of TNF-α levels in human PBMCs normalized against total protein. (**A**) TNF-α levels were measured in untreated human PBMCs, PBMCs treated with either 5 ng/mL spike protein (SP), 10 ng/mL SP, 40 mM liposomal GSH (L-GSH), 40 mM L-GSH plus 5 ng/mL SP, 40 mM L-GSH plus 10 ng/mL SP, 80 mM L-GSH, 80 mM L-GSH plus 5 ng/mL SP, or 80 mM L-GSH plus 10 ng/mL SP at 1 h post-treatment; (**B**) total TNF-α levels were measured in the same groups as Figure 5A at 3 days post-treatment; (**C**) total TNF-α levels were measured in the same groups as Figure 5A at 7 days post-treatment. Statistically significant *p*-values are indicated by asterisks, and *p*-values < 0.01 (**), <0.001 (***), and <0.0001 (****) are considered significant.

The relationship between TNF-α and spike protein was monitored in both treated and untreated PBMCs. A significant increase in TNF-α production was noted with increased spike protein in untreated samples on both day one (*p* < 0.01) and day three (*p* < 0.0001). When 5 ng/mL of spike protein was treated with 40 mM of L-GSH, a significant decrease was observed on day one (*p* < 0.0001), day three (*p* < 0.0001), and day seven (*p* < 0.001). When 5 ng/mL of spike protein was treated with 80 mM of L-GSH, a similar significant decrease was noted on day one (*p* < 0.0001), day three (*p* < 0.0001), and day seven (*p* < 0.001). The 10 ng/mL of spike protein was also treated with 40 mM of L-GSH producing significant decreases in TNF-α on day one (*p* < 0.0001), day three (*p* < 0.0001), and day seven (*p* < 0.0001). A similar decrease was noted when 10 ng/mL of spike protein was treated with 80 mM of L-GSH on day one (*p* < 0.0001), day three (*p* < 0.0001), and day seven (*p* < 0.0001) (Figure 5).

### 3.3. Liposomal Glutathione Supplementation Reduced Malondialdehyde Levels in Human Peripheral Blood Mononuclear Cells

**Figure 6:**
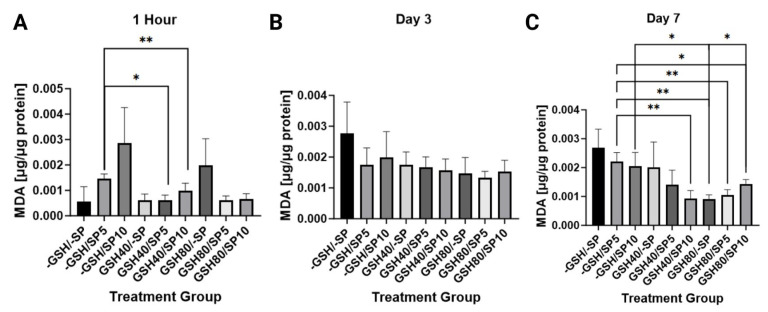
Measurement of malondialdehyde levels in human PBMCs normalized against total protein. (**A**) MDA levels were measured in untreated human PBMCs, PBMCs treated with either 5 ng/mL spike protein (SP), 10 ng/mL SP, 40 mM liposomal GSH (L-GSH), 40 mM L-GSH plus 5 ng/mL SP, 40 mM L-GSH plus 10 ng/mL SP, 80 mM L-GSH, 80 mM L-GSH plus 5 ng/mL SP, or 80 mM L-GSH plus 10 ng/mL SP at 1 h post-treatment; (**B**) total GSH levels were measured in the same groups as Figure 6A at 3 days post-treatment; (**C**) total GSH levels were measured in the same groups as Figure 6A at 7 days post-treatment. Statistically significant *p*-values are indicated by asterisks, and *p*-values < 0.05 (*) and <0.005 (**) are considered significant.

MDA is a lipid peroxidation byproduct and a marker for oxidative stress. MDA was measured between L-GSH- and spike protein-treated and untreated PBMCs. A 40 mM L-GSH treatment in those treated with 5 ng/mL of spike protein caused a significant decrease in MDA levels on day 1 (1 h) (*p* < 0.05) and day 7 (*p* < 0.005), with no statistically significant difference on day 3. A 40 mM L-GSH treatment in those treated with 10 ng/mL of spike protein caused a significant decrease in MDA levels on day 1 (1 h) and day 7 (*p* < 0.005), with no statistically significant difference on day 3. Additionally, an 80 mM L-GSH treatment in those treated with either no spike protein (*p* < 0.005), 5 ng/mL (*p* < 0.005), or10 ng/mL (*p* < 0.05) caused a significant decrease in MDA levels on day 7 when compared to those treatments with no L-GSH treatment and 5 ng/mL of spike protein. Furthermore, an 80 nM L-GSH treatment in those treated with either no spike protein (*p* < 0.05) or 10 ng/mL of spike protein (*p* < 0.05) caused a significant decrease in MDA levels on day 7 when compared to those with no L-GSH treatment and 10 ng/mL of spike protein (Figure 6).

### 3.4. Liposomal Glutathione Supplementation Reduced Microclot Formation

**Figure 7:**
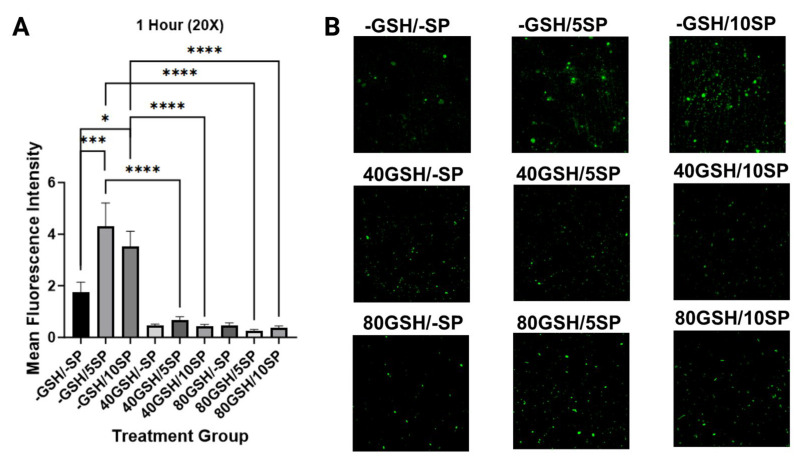
Measurement of mean thioflavin fluorescence intensity in human PBMC at 1 h. (**A**) Mean thioflavin T fluorescence intensity was measured in untreated human PBMCs, PBMCs treated with either 5 ng/mL spike protein (SP), 10 ng/mL SP, 40 mM liposomal GSH (L-GSH), 40 mM L-GSH plus 5 ng/mL SP, 40 mM L-GSH plus 10 ng/mL SP, 80 mM L-GSH, 80 mM L-GSH plus 5 ng/mL SP, or 80 mM L-GSH plus 10 ng/mL SP at 1 h post-treatment. Comparisons were made using one-way ANOVA. Statistically significant *p*-values are indicated by asterisks, and *p*-values < 0.05 (*), <0.001 (***), and <0.0001 (****) are considered significant. (**B**) Microscopic visualization of thioflavin T fluorescence intensity in untreated human PBMCs, PBMCs treated with either 5 ng/mL spike protein (SP), 10 ng/mL SP at 1 h post-treatment; microscopic visualization of thioflavin T fluorescence intensity in 40 mM liposomal GSH (L-GSH), 40 mM L-GSH plus 5 ng/mL SP, 40 mM L-GSH plus 10 ng/mL SP at 1 h post-treatment; microscopic visualization of thioflavin T fluorescence intensity in 80 mM L-GSH, 80 mM L-GSH plus 5 ng/mL SP, or 80 mM L-GSH plus 10 ng/mL SP at 1 h post-treatment.

**Figure 8:**
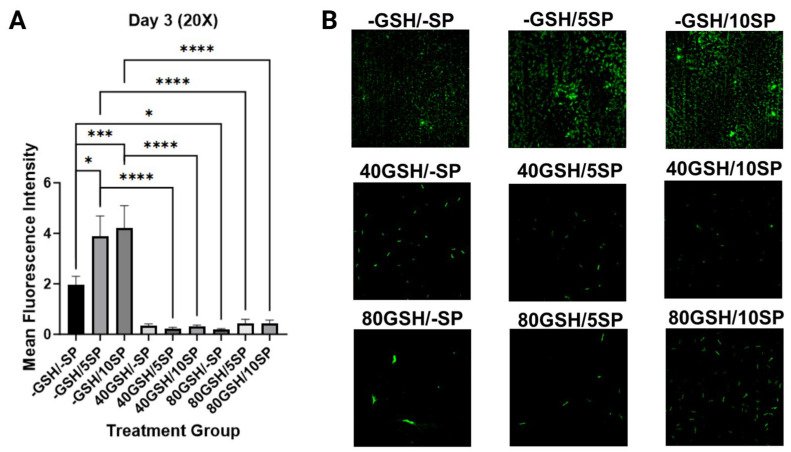
Measurement of mean thioflavin fluorescence intensity in human PBMC at day 3. (**A**) Mean thioflavin T fluorescence intensity was measured in untreated human PBMCs, PBMCs treated with either 5 ng/mL spike protein (SP), 10 ng/mL SP, 40 mM liposomal GSH (L-GSH), 40 mM L-GSH plus 5 ng/mL SP, 40 mM L-GSH plus 10 ng/mL SP, 80 mM L-GSH, 80 mM L-GSH plus 5 ng/mL SP, or 80 mM L-GSH plus 10 ng/mL SP at 3 days post-treatment. Comparisons were made using one-way ANOVA. Statistically significant *p*-values are indicated by asterisks, and *p*-values < 0.05 (*), <0.001 (***), and <0.0001 (****) are considered significant. (**B**) Microscopic visualization of thioflavin T fluorescence intensity in untreated human PBMCs, PBMCs treated with either 5 ng/mL spike protein (SP), 10 ng/mL SP at 3 days post-treatment; microscopic visualization of thioflavin T fluorescence intensity in 40 mM liposomal GSH (L-GSH), 40 mM L-GSH plus 5 ng/mL SP, 40 mM L-GSH plus 10 ng/mL SP at 3 days post-treatment; microscopic visualization of thioflavin T fluorescence intensity in 80 mM L-GSH, 80 mM L-GSH plus 5 ng/mL SP, or 80 mM L-GSH plus 10 ng/mL SP at 3 days post-treatment.

**Figure 9:**
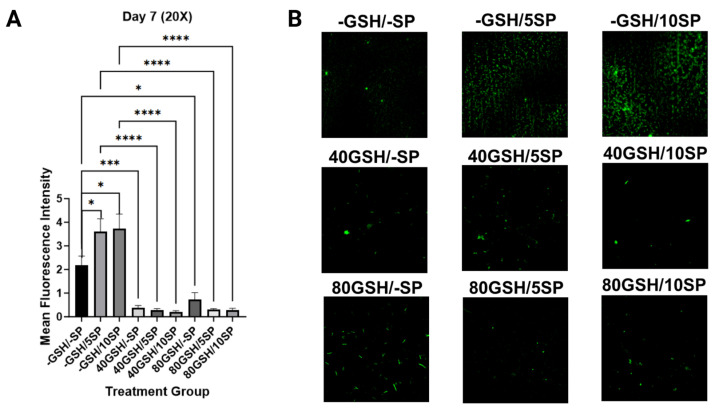
Measurement of mean thioflavin fluorescence intensity in human PBMC at day 7. (**A**) Mean thioflavin T fluorescence intensity was measured in untreated human PBMCs, PBMCs treated with either 5 ng/mL spike protein (SP), 10 ng/mL SP, 40 mM liposomal GSH (L-GSH), 40 mM L-GSH plus 5 ng/mL SP, 40 mM L-GSH plus 10 ng/mL SP, 80 mM L-GSH, 80 mM L-GSH plus 5 ng/mL SP, or 80 mM L-GSH plus 10 ng/mL SP at 7 days post-treatment. Comparisons were made using one-way ANOVA. Statistically significant *p*-values are indicated by asterisks, and *p*-values < 0.05 (*), <0.001 (***), and <0.0001 (****) are considered significant. (**B**) Microscopic visualization of thioflavin T fluorescence intensity in untreated human PBMCs, PBMCs treated with either 5 ng/mL spike protein (SP), 10 ng/mL SP at 7 days post-treatment; microscopic visualization of thioflavin T fluorescence intensity in 40 mM liposomal GSH (L-GSH), 40 mM L-GSH plus 5 ng/mL SP, 40 mM L-GSH plus 10 ng/mL SP at 7 days post-treatment; microscopic visualization of thioflavin T fluorescence intensity in 80 mM L-GSH, 80 mM L-GSH plus 5 ng/mL SP, or 80 mM L-GSH plus 10 ng/mL SP at 7 days post-treatment.

To quantify microclot formation, microscopic visualization and measurement of thioflavin fluorescence were performed at 1 h, 3-day, and 7-day intervals. A significant increase in thioflavin T fluorescence was observed with the addition of spike protein to untreated PBMC samples, examining 5 ng/mL SP-PBMC samples at one hour (*p* < 0.001), day three (*p* < 0.05), and day seven (*p* < 0.05), and examining 10 ng/mL SP-PBMC samples at one hour (*p* < 0.05), day three (*p* < 0.001), and day seven (*p* < 0.05). When 5 ng/mL SP-PBMC samples were treated with 40 mM of L-GSH, a significant decrease in fluorescence was observed at one hour (*p* < 0.0001), day three (*p* < 0.0001), and day seven (*p* < 0.0001). When 5 ng/mL SP-PBMC samples were treated with 80 mM of L-GSH, a significant decrease in fluorescence was noted at one hour (*p* < 0.0001), day three (*p* < 0.0001), and day seven (*p* < 0.0001). Similarly, when 10 ng/mL of spike protein-PBMC samples were treated with 40 mM of L-GSH, a significant decrease in thioflavin fluorescence was registered at one hour (*p* < 0.0001), day three (*p* < 0.0001), and day seven (*p* < 0.0001). A similar decrease was noted when 10 ng/mL of spike protein-PBMC samples was treated with 80 mM of L-GSH at one hour (*p* < 0.0001), day three (*p* < 0.0001), and day seven (*p* < 0.0001). Lastly, we observed a decrease in total thioflavin fluorescence in spike protein-free PBMC samples when treated with 40 mM and 80 mM on days three and seven, with (*p* < 0.001) and (*p* < 0.05) achieved consecutively (Figure 7, Figure 8 and Figure 9).

## 4. Discussion

As of 7 January 2024, 774 million cases of COVID-19 and another 7 million deaths have been reported across the globe [29]. Even 4 years after the initial outbreak of the disease, COVID-19 continues to remain a major health concern globally. While vaccinations have been used preventatively in COVID-19, and other therapies exist to treat active infection, including antivirals, and while vaccinations and treatment can reduce a patient’s chances of both contracting the disease as well as developing complications such as acute respiratory distress syndrome, patients can still contract the disease and experience poor outcomes. As such, the discovery of novel methods that can reduce morbidity and mortality in COVID-19 patients is an important area of research. In lieu of a live COVID-19 virus, this study investigated the utility of a novel formulation of L-GSH in PBMC cultures utilizing the SARS-CoV-2 spike protein. The results of our study demonstrate that L-GSH supplementation can reduce adverse factors of the spike protein within a PBMC in vitro model, which is an important first step in the evaluation of L-GSH in COVID-19.

In order for GSH to have an effect upon intracellular metabolism, it must first penetrate the cell membrane. The GSH supplement was formulated within liposomes (L-GSH) to increase the penetrative capability of the supplement. The function of L-GSH’s ability to penetrate the cell membrane and its influence on the cells’ GSH production was evaluated through the GSH assays conducted using the cell culture pellets at three timepoints of 1 h, 3 days, and 7 days. Our study found that the detectable levels of GSH within the cell pellets were significantly increased in the cell cultures treated with glutathione regardless of the concentration of glutathione treatment, indicating that L-GSH was able to penetrate the cell membrane. The cells treated with L-GSH may also have synthesized additional endogenous GSH since the cytokines IL-6 and TGF-β are known to decrease GSH synthesis and these cytokines were found to be decreased in the cultures treated with L-GSH [12,13,15,16], (Figure 3 and Figure 4). Previous studies have also found that GSH levels are decreased in COVID-19 [25]. The low, albeit detectable, levels of GSH within the cells untreated with our L-GSH treatment indicate that the cells in this control group at least produced their own endogenous GSH. It is also notable that there was no significant difference in detectable GSH levels between spike protein concentrations. This could suggest that, even though GSH levels are decreased in COVID-19, the spike protein treatment alone, in the absence of a live virus, may be insufficient to induce a change in GSH levels in the cell cultures.

Previous studies have found that the SARS-Cov-2 spike protein is associated with increased oxidative stress and reactive oxygen species within PBMCs [30]. Increased oxidative stress has also been found to be an important factor in COVID-19 infection and has even been shown to be involved with virus replication [31]. GSH is an antioxidant molecule that reduces oxidative stress on many cellular components [32]. Within the body, the enzyme glutathione peroxidase 3 (GPX-3) utilizes GSH to decrease the amounts of reactive oxygen species (ROS) [29]. The antioxidant ability of GSH and GPX-3 has also been suggested to aid in the prevention of clot formation through their reduction of ROS and nitric oxide species since these are thought to increase clot formation by modifying fibrinogen [33]. With GSH’s role as an antioxidant, we hypothesized that L-GSH would reduce reactive oxygen species and oxidative stress within the cell cultures, both those treated with spike protein and those treated without. Our results in the MDA assays found significant reductions in oxidative stress in the L-GSH-treated cell cultures at the one-hour and seven-day timepoints, demonstrating that L-GSH can reduce spike protein-induced oxidative stress in vitro. Since ROS are thought to play a role in clot formation, they may also play an important role in COVID-19 immunothrombosis. If L-GSH supplementation is shown to have the same effect of reducing oxidative stress in vivo, this would suggest that L-GSH may have potential as an additional therapy for COVID-19 due to its reduction of ROS, which may aid in the prevention of immunothrombosis.

IL-6, TGF-beta, and TNF-alpha are all cytokines that have been found to have connections with GSH and COVID-19. Studies have associated the disease with increased levels of IL-6 and TNF-alpha [34]. TNF-alpha has also been shown to play a vital role in inflammatory cell death in COVID-19. Studies have also found that increased IL-6 levels are associated with tissue injury, hyperinflammation, and specifically more severe disease and progression to acute respiratory failure in COVID-19 [35,36,37]. Thus, IL-6 and TNF-alpha have all been shown to be associated with the disease process. In addition, previous studies have indicated that IL-6 and TGF-beta can decrease GSH levels by lowering the activity of glutamate cysteine ligase [12,13,15,16]. Since IL-6 and TNF-alpha are inflammatory cytokines that increase ROS, their upregulation in COVID-19 leads to increasingly depleted GSH and increasingly high ROS. Previous papers have suggested that this process both damages tissues directly and also contribute to microthrombosis by dramatically increasing ROS, which in turn modifies fibrinogen [38]. Due to the central role that GSH appears to play in COVID-19, we hypothesized that supplementing endogenous glutathione with L-GSH would interrupt this cycle, which could be evidenced by decreased levels of the previously mentioned cytokines due to reduced ROS. In the experiment, our results seem to confirm the aforementioned process by showing an overall decrease in the levels of IL-6, TGF-β, and TNF-α when cultures were treated with L-GSH (Figure 3, Figure 4 and Figure 5). We also found that spike protein concentration was directly correlated with IL-6 and TNF alpha levels in some of the samples. One interpretation of these results is that supplemental L-GSH restores cytokine balance by lowering ROS directly and by interrupting the previously mentioned cycle of continuous GSH depletion. Our results show that L-GSH lowered TGF-beta and IL-6. Interestingly, they also suggest that GSH supplementation may be able to induce its own endogenous synthesis by removing the inhibition of glutamate cysteine ligase. The results also align with a previous study from our own lab demonstrating that GSH supplementation reduces the production of IL-6 and TGF-beta in HIV patients, indicating that a similar process may be occurring between both diseases [39].

GSH has been suggested as a means to decrease clot formation in COVID-19 due to its ability to reduce ROS and NO species, which can cause clot formation by modifying fibrinogen, as previously mentioned [38]. Previous studies have also found that knockout mice for GXP-3, the enzyme that uses GSH to lower ROS, demonstrate increased platelet-induced microthrombosis [29]. GSH also can reduce fibrin clot formation by assisting macrophages in removing fibrin [40]. With immunothrombosis being one of the proposed mechanisms in COVID-19 infection, we wanted to investigate this process and hypothesized that L-GSH supplementation would reduce fibrin clot formation within the PBMC cultures by reducing ROS and assisting in fibrin clearance. The results of the thioflavin fluorescence study found that L-GSH supplementation was associated with decreased fluorescence intensity and thus decreased fibrin clot formation, while spike protein treatment was associated with increased fluorescence and fibrin clot formation (Figure 7, Figure 8 and Figure 9). While other studies have found that COVID-19 infection is associated with decreased GSH and increased immunothrombotic clot formation, this is the first study to directly suggest that L-GSH can reduce the formation of fibrin clots induced by the COVID-19 spike protein [1,2,35]. Since immunothrombosis is one of the factors associated with acute respiratory distress syndrome, the results imply that L-GSH supplementation may be effective in reducing the mortality and morbidity associated with severe COVID-19 infection, but future studies are needed to investigate this [1,2].

While our study found that GSH supplementation is effective in reducing the effects of SARS-CoV-2 spike protein on PBMCs in vitro, the study does have important limitations. Firstly, the study was conducted exclusively within a cell culture model. For clinical conclusions to be made with regard to L-GSH supplementation within patients with COVID-19, studies will have to be conducted in a clinical trial model with live patients. Thus, while this study can offer conclusions about L-GSH and spike protein effects in a cell culture model, no conclusions can be made about L-GSH effects on patients infected with COVID-19. It is also possible that the concentrations of L-GSH used in this experiment may not be possible to achieve in a human. Further studies must be conducted to determine if L-GSH can be used to reduce morbidity and mortality in the disease. Another limitation of our study is that all experiments were conducted using only PBMCs. While PBMCs are important in the pathogenesis of disease, other cells are likely involved in the pathogenesis of COVID-19. In particular, this study focused on cytokines, which are produced by many cells but especially by T cells. Conducting a similar study, using T cells and other blood cells in addition to PBMCs, may yield additional insights. Another limitation is that all these studies were conducted using spike protein rather than a live virus for practical reasons. While studies have suggested that spike protein by itself can cause many effects similar to those of the virus, future studies involving the full virus should be conducted to better assess the effects of L-GSH on COVID-19. There was also a limitation in the data regarding the MDA data for day 3. For reasons that are not clear, the day 3 timepoint for the MDA studies demonstrated no statistical significance, which was resolved upon day seven. It is possible that the cell cultures may have experienced an unknown variable which affected their oxidative stress levels. Further investigation should be conducted into this phenomenon. Finally, the study was also limited by the small sample size of individuals. While this study was conducted with 9 volunteers, it would be useful to conduct future studies with additional participants to better account for the variations in individuals’ immune systems.

## 5. Conclusions

This study has demonstrated the efficacy of L-GSH within a cell culture model using high concentrations of L-GSH supplements. It represents a vital first step in investigating treatment with GSH, a new potential therapy for COVID-19. The study found that L-GSH increases intracellular total GSH, decreases IL-6, TGF-beta and TNF-alpha levels, decreases oxidative stress, and decreases fibrin clot formation in untreated PBMC cell cultures and in those treated with SARS CoV-2 spike protein. All these factors are believed to be involved in COVID-19 disease and contribute to the development of acute respiratory distress syndrome. While this study demonstrates the ability to reduce these factors in a cell culture model, more research is needed to determine if L-GSH is an effective supplement for reducing the morbidity and mortality of patients with COVID-19. With the efficacy of L-GSH demonstrated in vitro, GSH shows great promise for future investigations into new COVID-19 treatments.

## Data Availability

The data supporting reported results can be obtained from the corresponding author (V.V.) upon formal requisition.
